# The concomitant assessment of pain and dyspnea in acute exacerbations of chronic obstructive pulmonary disease; is pain an understudied factor?

**DOI:** 10.1177/14799731221105516

**Published:** 2022-06-14

**Authors:** Emily Hume

**Affiliations:** 1 Department of Sport, Exercise and Rehabilitation, Faculty of Health & Life Sciences, 373117 Northumbria University, Newcastle upon Tyne, UK

Dyspnea is a prominent symptom of Chronic Obstructive Pulmonary Disease (COPD), occurring as a result of expiratory flow limitation, which may lead to varying degrees of dynamic hyperinflation, hypoxemia, hypercapnia and neuromechanical dissociation.^1^ Although pain appears to be a prevalent symptom in COPD patients,^2^ it is rarely considered in clinical practice guidelines for the management of the disease,^3^ which could be due to pain being a complex and understudied factor in COPD. When compared to other disease entities (diabetes, heart disease and arthritis), COPD patients had an increased risk of pain prevalence and intensity, second only to those with arthritis.^4^ There are several underlying mechanisms that may contribute to higher pain prevalence in COPD compared to healthy individuals. These include increased and persistent respiratory muscle loading, along with systemic inflammation, musculoskeletal disorders and co-morbities.^5^ In patients with stable COPD, pain is a prevalent symptom which negatively impacts quality of life,^2^ and is associated with higher levels of lung hyperinflation and dyspnea.^6,7^ An acute exacerbation of COPD (AECOPD) occurs when there is an acute worsening of respiratory symptoms requiring additional treatment.^8^ Thus, given the relationship between the two perceptions, many factors linked to pain in the stable state tend to worsen during an AECOPD.

The systematic review by Clarke et al. focused on the prevalence of pain and dyspnea experienced concurrently in people admitted to hospital with an AECOPD. A total of 1300 articles were identified from initial database searches, however only four studies met the inclusion criteria and were included in the review. Pain and dyspnea are both unpleasant sensations which share many clinical, physiological and psychological features.^9^ Brain imaging studies highlight that perceptions of pain and dyspnea activate similar cortical regions of the brain,^10^ and share common neural mechanisms.^11^ Due to these commonalities, the review aimed to further understand the interactions between pain and dyspnea, and their clinical implications during an AECOPD. Of the available data, pooled prevalence of pain and dyspnea was 44% and 91% respectively, demonstrating that both symptoms are prevalent in COPD patients during acute exacerbations. However, due to the small number of studies co-reporting pain and dyspnea, the scope of the review to draw clinical associations and implications of both symptoms during AECOPD was limited.

As described by the authors in the review, management of COPD exacerbations primarily focuses on relieving dyspnea, reducing medication and oxygen requirements, returning to baseline function and follow up care. Discharge care bundles have been shown to reduce hospital re-admissions following hospitalisation for an AECOPD, but did not improve survival or quality of life.^12^ The individual components of discharge bundles tend to vary and it is not clear whether pain is considered within the education and self-management plans. This is likely due to pain during exacerbations being under recognised and under researched, as highlighted by Clarke et al., limiting the evidence base for including pain management in clinical care guidelines. However, it is important to distinguish published research from frontline clinical care, as clinical providers may be treating symptoms such as pain routinely and detailing this in patient records, which may not be captured in a research study.

The research examining pain independently of dyspnea during AECOPD still appears to be limited. Cheng et al.^13^ showed that pain intensity and interference scores were significantly higher during AECOPD, compared to stable COPD. However, there was no significant difference in pain prevalence between AECOPD (31% of patients) and stable COPD (24% of patients), or pain locations (chest and back). This finding differs from the study by Maignan et al.^7^ who showed pain was usually located in the limbs during the stable phase, but in the chest during an AECOPD. Pain in the back, shoulders, neck and chest in COPD patients could be due to the excessive work of breathing, chest tightness and the perception of the urge to breath.^14^

In people with stable COPD, chronic pain has been shown to have numerous clinical implications, such as increased medical costs, as well as adversely impacting quality of life, fatigue, depression and participation in physical activity and activities of daily living.^3^ All of which are factors known to impact survival and clinical outcomes in COPD patients.^15,16^ Pain may also limit a patient’s ability to partake in evidence-based interventions such as pulmonary rehabilitation,^3^ which may contribute to the poor uptake and completion of this intervention.^17^ This indicates that pain warrants clinical attention in both stable COPD and AECOPD in routine practice, to better understand causes, trajectories and characteristics of pain.

Determining the optimal tool for assessing pain appears to be an important issue. The review by Clarke et al. highlights a wide range of different outcome measures and focal periods that have been used to assess pain and dyspnea, with six tools used for pain and four for dyspnea within the four studies included. This makes it challenging to synthesise information from the available evidence. A systematic review in stable COPD patients also highlighted this issue, with the most common tool being a pain specific questionnaire (Brief Pain Inventory), however some studies used a screening question (e.g., ‘Are you generally bothered with pain?’) or pain as a sub-domain of a quality of life instrument (e.g., SF-36 or EQ-5D).^2^ Thus, Clarke et al. importantly emphasise that standardising assessment tools with clearly defined focal periods is vital for improving understanding into the prevalence, intensity, and associations of pain.

In conclusion, the review from Clarke et al. highlights that dyspnea and pain are prevalent symptoms during AECOPD, which aligns with previous research examining these symptoms in stable COPD. Overall, the research evaluating pain as a symptom in COPD patients is limited, particularly during AECOPD. This is concerning due to its association with other symptoms, co-morbities and diminished quality of life. Thus, more research using standardised assessment tools is needed to better understand the causes, trajectories, and characteristics of pain independently of other symptoms in both stable and exacerbated COPD patients, and enable the development of effective treatment strategies.
